# Assessment of combined expression of B7-H3 and B7-H4 as prognostic marker in esophageal cancer patients

**DOI:** 10.18632/oncotarget.12628

**Published:** 2016-10-13

**Authors:** Lujun Chen, Quanqin Xie, Zhigang Wang, Liangrong Shi, Changping Wu, Jingting Jiang

**Affiliations:** ^1^ Department of Tumor Biological Treatment, The Third Affiliated Hospital of Soochow University, Changzhou, Jiangsu 213003, China; ^2^ Research Center for Cancer Immunotherapy Technology of Jiangsu Province, The Third Affiliated Hospital of Soochow University, Changzhou, Jiangsu 213003, China; ^3^ Department of Respiratory Medicine, The Third Affiliated Hospital of Soochow University, Changzhou, Jiangsu 213003, China; ^4^ Department of Oncology, The Third Affiliated Hospital of Soochow University, Changzhou, Jiangsu 213003, China; ^5^ Institute of Cell Therapy, The Third Affiliated Hospital of Soochow University, Changzhou, Jiangsu 213003, China

**Keywords:** B7-H3, B7-H4, immunohistochemistry, prognosis

## Abstract

The co-stimulatory ligands of B7-family have been confirmed to play an important role in negatively regulating the T-cell mediated anti-tumor immunity. In addition, these inhibitory molecules are also aberrantly expressed on various human cancers tissues, and significantly associated with cancer progression and patients' poor prognoses. We have previously reported that B7-H3 and B7-H4 ligands are highly expressed in human esophageal cancer tissues. Herein, we tried to further analyze the value of their combined expression on prognostic prediction for esophageal cancer patients. We found that the combined expression of both B7-H3 and B7-H4 could be used as a valuable risk factor for predicting the prognosis of esophageal cancer patients (*P*=0.003). Moreover the status of these patients with high expression of both B7-H3 and B7-H4, was positively and significantly associated with the tumor invasion depth (*P*=0.0414) and TNM stage (*P*=0.0414). The Cox multivariate proportional hazards regression analysis revealed that the tumor size (*P*=0.007), the TNM stage (*P*=0.024) and the status of both B7-H3 and B7-H4 high expression (*P*=0.011), could be used as an independent risk factor for predicting patients' postoperative prognosis, respectively. In conclusion, our data indicated that the combined application of B7-H3 and B7-H4 expression can be effectively used as a prognostic marker in esophageal cancer patients.

## INTRODUCTION

Esophageal cancer is one of the most important malignancies of human digestive tract, and still remains a significant problem in Chinese population [1]. Numerous factors such as alcohol intake, smoking, local inflammation, esophagitis, and increased cell turnover, has been shown to be involved in the complicated pathogenesis of esophageal cancer, and finally contribute to its progression [2]. Despite the use of series of therapeutic strategies such surgery, chemotherapy, radiotherapy, immunotherapy and even combined therapies in the clinical treatment of this malignancy, the 5-year survival still remains poor [3, 4].

The co-stimulatory family members, such as B7-H1, B7-H3, and B7-H4 have been shown to play an important role not only in the activation of T cells but also in the regulation of T-cell mediated anti-tumor response [5]. Additional data have indicated that these co-stimulatory molecules are also expressed by tumor cells, where they not only suppress T-cell mediated anti-tumor response, but also regulate the biological features of the tumor cells and thus contribute to the cancer initiation and progression [5, 6]. In our previous publications, we have demonstrated that these members of B7 family co-stimulators are aberrantly expressed in human esophageal cancer tissues, and could be used as valuable prognostic predictors [5, 7, 8]. Thus, it's now widely accepted that these molecules are involved in T-cell mediated tumor surveillance, have an important value for clinical application in addition to their use as critical immunotherapeutic targets [9].

In the present study, we focused on analyzing the possibility of using combined B7-H3 and B7-H4 expression, as a prognostic tool for predicting esophageal cancer patient's prognosis. Thus we have analyzed the expression of both B7-H3 and B7-H4 protein on the esophageal tumor tissue samples and correlated the expression with the clinical characteristics of these patients.

## RESULTS

### Analysis of B7-H3 and B7-H4 immunostaining in human esophageal cancer tissues

**Figure 1:**
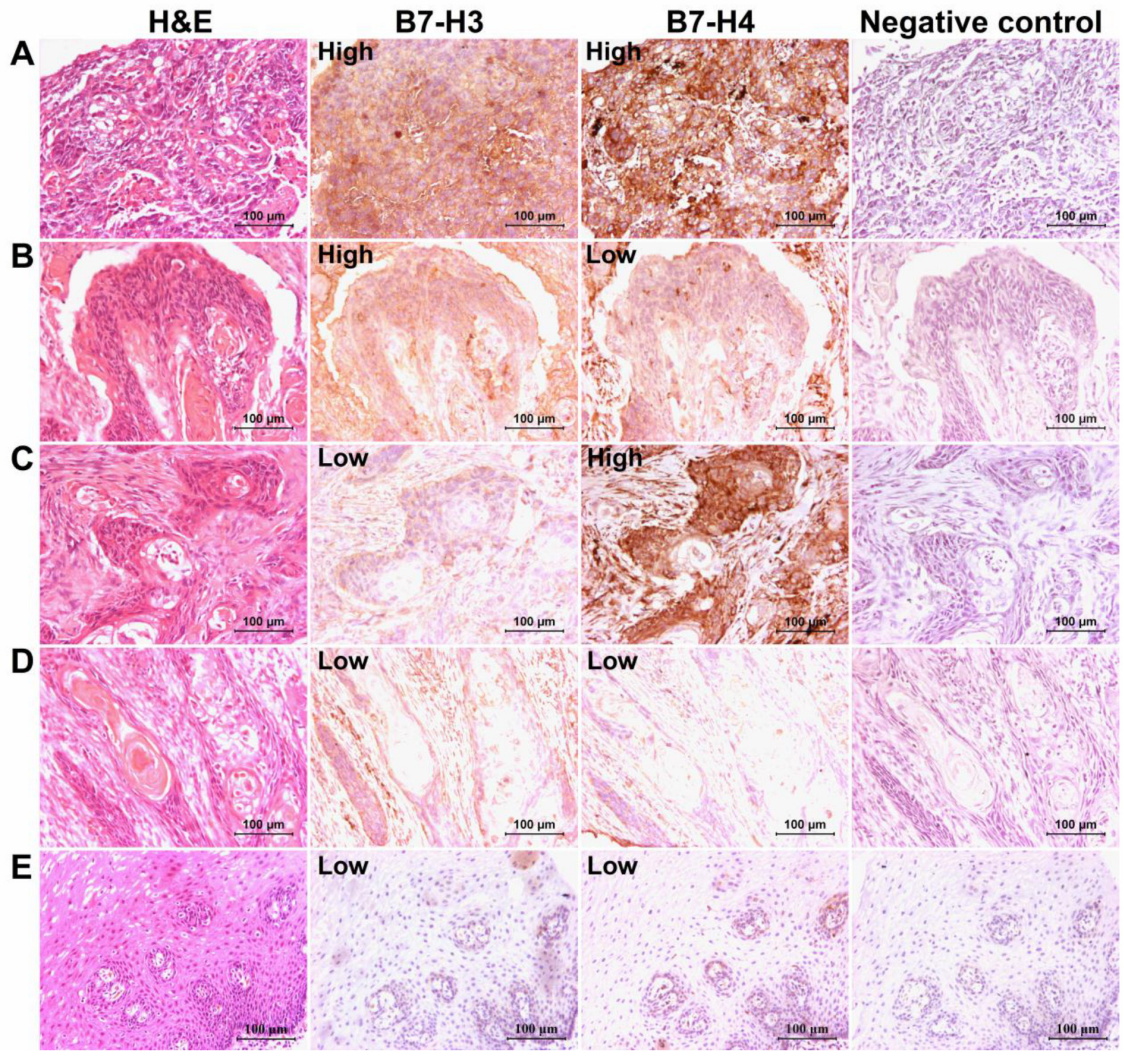
B7-H3 and B7-H4 immunostaining in human esophageal cancer tissues The panel **A.** represented the high expression of both B7-H3 and B7-H4 proteins, while panel **B.** showed, B7-H3 high and B7-H4 low expression, and panel **C.** revealed B7-H3 low and B7-H4 high expression. The panel **D.** represented the low expression of both B7-H3 and B7-H4 proteins. The panel **E.** represented the weak expression of B7-H3 as well as B7-H4 in adjacent normal esophageal tissues. (Scale bar = 100 μm).

The immunohistochemistry analysis showed that positive expression of B7-H3 and B7-H4 was predominantly on the membrane and in the cytoplasm of esophageal cancer cells (Figure 1A). In order to further investigate the correlation between clinical parameters and the combined expression of B7-H3 and B7-H4 in the esophageal cancer tissues, the 103 patients were categorized into two subgroups according to the staining intensity (*H-score*) of B7-H3 (cut-off value = 125), and B7-H4 (cut-off value = 160). Based on this criterion, 51 patients were observed to have high expression of both B7-H3 and B7-H4 proteins. Moreover, the adjacent normal esophageal cancer tissues were used as control, and we found that both B7-H3 and B7-H4 were weakly expressed on esophageal epithelial cells of normal tissues (Figure 1E).

### Analysis of the clinical implications and prognostic predictability of B7-H3 and B7-H4 combined expression in esophageal cancer patients

**Figure 2:**
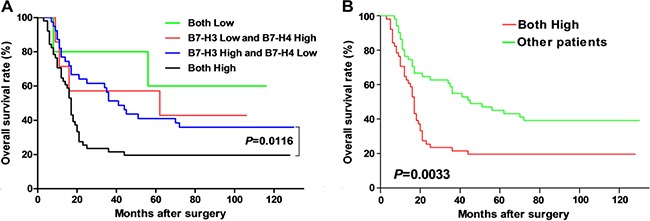
Correlation analysis between different expression of B7-H3 and B7-H4 proteins and overall survival in esophageal cancer patients Panel **A.** showed that comparison of the overall survival rate of the patients with B7-H3 high and B7-H4 low vs. patients with both B7-H3 and B7-H4 high expression. Panel **B.** showed the comparison of the overall survival rate of the patients with both B7-H3 and B7-H4 high expression *vs.* all the other patients.

The subgroup analysis of B7-H3 and B7-H4 high expression based on gender revealed that significantly more number of females have high expression of both these proteins together than males (*P*=0.0229). Moreover, the T_2+3+4_ subgroup also showed significantly high expression of both these proteins than T_1_subgroup (*P*=0.0414). Further subgroup analysis based on the TNM stage indicated that patients with TNM stage (II+III+IV), have significantly high expression of both these proteins than patients from TNM stage (I) subgroup (*P*=0.0414). All these subgroup analysis have been shown in Table 1. Next, the survival analysis demonstrated that the tumor size, TNM stage, and the status of the combined B7-H3 and B7-H4 expression levels, were all significantly associated with patients' postoperative prognoses (Figure 2A and 2B). The Cox regression analysis model also showed that all these three factors could be used as independent risk predictor respectively (Table 2).

**Table 1:** Correlation between the clinical parameters and B7-H3 expression in combination with B7-H4 expression in esophageal cancer tissues

Clinical parameters	Cases	Combination of B7-H3 and B7-H4 expression			*P*-value
		Both high (%)	Other patients (%)	*χ*^2^	
Gender					
Male	75	32 (42.67)	43 (57.33)	5.176	**0.0229**
Female	28	19 (67.86)	9 (32.14)		
Age (years)					
< 60	63	34 (53.97)	29 (46.03)	1.287	0.2566
≥ 60	40	17 (42.50)	23 (57.50)		
Tumor size (cm)					
< 3.5	38	18 (47.37)	20 (52.63)	0.1109	0.7391
≥ 3.5	65	33 (50.77)	32 (49.23)		
Depth of invasion (T)					
T_1_	13	3 (23.08)	10 (76.92)	4.160	**0.0414**
T_2+3+4_	90	48 (53.33)	42 (46.67)		
Nodal metastasis (N)					
Yes	49	29 (59.18)	20 (40.82)	3.496	0.0615
No	54	22 (40.74)	32 (59.26)		
Distant metastasis (M)					
Yes	16	11 (68.75)	5 (31.25)	2.804	0.0940
No	87	40 (45.98)	47 (54.02)		
TNM stage					
I	13	3 (23.08)	10 (76.92)	4.160	**0.0414**
II+III+IV	90	48 (53.33)	42 (46.67)		

Values in bold signify *P* < 0.05

**Table 2:** Prognostic factors analysis based on Cox's proportional hazards model

Clinical parameters	Uni-variate			Multi-variate		
	Hazard ratio	95% CI	*P*-value	Hazard ratio	95% CI	*P*-value
Gender						
Male / Female	0.780	0.473-1.286	0.329	0.898	0.553-1.515	0.688
Age (years)						
≥ 60 / < 60	1.100	0.688-1.759	0.690	1.038	0.638-1.689	0.880
Tumor size (cm)						
≥ 3.5 / < 3.5	2.213	1.319-3.711	**0.003**	2.101	1.228-3.596	**0.007**
TNM Stage						
S_III+IV_ / S_I+II_	1.886	1.168-3.044	**0.009**	1.758	1.079-2.864	**0.024**
B7-H3 and -H4 combined						
Both high / others	2.066	1.272-3.355	**0.003**	1.876	1.155-3.046	**0.011**

Values in bold signify *P* < 0.05.

## DISCUSSION

Many members from the B7 family ligands not only contribute towards the regulation of T-cell mediated immune response, but also have important implications in the regulation of the biological behaviors of cancer cells, leading to cancer progression and metastasis [10–12]. We have previously identified that B7-H1 expression in human esophageal cancer was significantly associated with tumor invasion and patient's prognosis [13]. In addition, B7-H4 expression in human gastric cancer and esophageal cancer have also been suggested to be significantly associated with cancer progression, reduced tumor immuno-surveillance as well as worse patient outcomes, and thus the role of B7-H4 molecule as a novel prognostic predictor for these malignancies has been predicted [4, 14]. We also have reported a significant correlation between the expression levels of both B7-H4 and B7-H1 in human gastric cancer, and significant association with poor prognosis [15]. Similarly, Chen *et al*. [16] also reported that B7-H1, B7-H3, and B7-H4 molecules are involved in pancreatic cancer progression, and indicated that their co-expression could be used as a valuable prognostic marker, and can have important clinical application in predicting cancer patients' prognosis.

Recently, we showed that high B7-H3 expression was linked with cancer progression of human esophageal cancer, and can negatively regulate T-cell mediated anti-tumor response in the tumor microenvironment, proliferation and the mobility of the esophageal cancer cells [17]. However, the clinical implications and the potential prognostic value of the combination of these biomarkers in human esophageal cancer still remain elusive, and we have tried to address this issue in our current study. B7-H3 not only has an immunological role in anti-tumor response, but also contributed essentially in the promotion of tumor invasion, metastasis, and drug resistance of the cancer cells [18–22]. The detailed analysis of its mechanism revealed that PI3K/AKT/mTOR pathways seems to be involved in B7-H3 mediated regulation of the biological behaviors of cancer cells [19]. The other member, B7-H4 was also suggested to be an important risk factor in predicting cancer patient's prognosis [4, 5, 23–27]. Both the membrane and soluble forms of B7-H4 in cancer cells and in peripheral circulation, respectively, are valuable biomarkers in human cancers [28–30]. B7-H4 was also involved in the regulation of cancer cell behaviors such as proliferation, cell cycle arrest, migration and invasion. The gene set enrichment analysis showed that CXCL12/CXCR4 and JAK/STAT pathways were correlated with the B7-H4 expression [31].

In present study, found that patients with high expression of both B7-H3 and B7-H4 tended to have increased invasion as well as high TNM stage. Moreover, we identified that the combination of B7-H3 and B7-H4 expression could be used as a valuable risk factor for predicting the prognoses of esophageal cancer patients. The multivariate proportional hazards regression analysis further revealed that the tumor size, TNM stage as well as high expression of both B7-H3 and B7-H4, could be used as an independent risk factor for predicting patients' postoperative prognoses. Thus, our data suggested that the assessment of combined expression of B7-H3 and B7-H4 can be effectively used as prognostic predictor in esophageal cancer patients.

## MATERIALS AND METHODS

### Patients and tissues samples

Formalin-fixed, paraffin-embedded esophageal cancer tissue samples were collected from 103 patients (75 men and 28 women; median age at diagnosis was 58 years), who underwent surgical resection between December 2000 and March 2005 in our hospital. In addition, 5 normal tissues were collected from the non-malignant portion of esophagus during surgery to be used as controls. None of the patients received pre-operative chemotherapy or radiotherapy. All the tumor tissues were confirmed as esophageal squamous cell carcinoma after surgical resection, by using hematoxylin and eosin (H&E) staining. The detailed clinical parameters of the patients are shown in Table 1. The protocols for this study were approved by the ethics committee of the hospital.

### Antibodies

Goat anti-human B7-H3 polyclonal antibody (AF1027) was purchased from R&D Systems, Inc (Minneapolis, MN, USA). Rabbit anti-human B7-H4 polyclonal antibody (NBP2-30536) was purchased from Novus Biologicals (Littleton, CO, USA). HRP-labeled goat anti mouse/rabbit secondary antibody (K500711, ready to use) was purchased from Dako (Glostrup, Denmark). Polink-2 plus^®^ polymer HRP detection system for goat primary antibody (PV-9003) was obtained from Zhongshan Golden Bridge Biology (Beijing, China).

### Immunohistochemistry

Formalin-fixed, paraffin-embedded tissues were cut into 3-μm-thick consecutive sections, and were dewaxed in xylene, rehydrated and graded in ethanol solutions. Antigens were retrieved by heating the tissue sections at 100 °C for 30 min in EDTA (1mmol/L, pH9.0) (for B7-H3) or in citrate (10mmol/L, pH6.0) (for B7-H4) solution, when needed. The sections were then immersed in a 0.3% hydrogen peroxide solution for 30 min to block endogenous peroxidase activity, rinsed in phosphate buffered saline (PBS) for 5 min, blocked with 3% BSA at room temperature for 30 min, and finally incubated with purified goat anti-human B7-H3 (2.5μg/ml), or rabbit anti-human B7-H4 antibody (1:1000 dilution) at 4 °C overnight. A negative control was processed by omitting the primary antibody. The Polink-2 plus^®^ polymer HRP detection system for goat primary antibody, or the HRP-labeled goat against rabbit secondary antibody, was used according to the manufacture's instruction. Later, the diaminobenzene was used as a chromogen, and hematoxylin as the nuclear counterstain. The sections were then dehydrated, cleared and mounted.

### Evaluation of immunohistochemical staining

All slides were examined independently by two senior pathologists, who were not informed of patients' clinical parameters. The B7-H3 as well as B7-H4 immunostaining densities were assessed according to the *H-score* method which has been described by previously [32, 33]: *H-score* = (% tumor cells unstained x0) + (% tumor cells stained weak x1) + (% tumor cells stained moderate x2) + (% tumor cells stained strong x3). The *H-scores* ranged from 0 (100% negative staining) to 300 (100% strong staining). The results obtained from two pathologists from the five areas, were averaged and statistically analyzed.

### Statistical analysis

Statistical analysis was performed by using the GraphPad Prism 5.0 software package (GraphPad Software, Inc., San Diego, USA). The Chi-square test or the survival analysis was used where appropriate. A *p*-value of <0.05 was deemed significant.
